# Aberrant O-glycosylation modulates aggressiveness in neuroblastoma

**DOI:** 10.18632/oncotarget.26169

**Published:** 2018-09-25

**Authors:** Hector A. Cuello, Valeria I. Segatori, Marina Albertó, Cynthia A. Gulino, Rosario Aschero, Sandra Camarero, Laura Galluzzo Mutti, Kevin Madauss, Daniel F. Alonso, Fabiana Lubieniecki, Mariano R. Gabri

**Affiliations:** ^1^ Molecular Oncology Laboratory, Quilmes National University, Bernal, Buenos Aires Province, Argentina; ^2^ Department of Pathology, Pediatric Hospital “Prof. Dr. Juan P. Garrahan”, Buenos Aires, Argentina; ^3^ GlaxoSmithKline, Philadelphia, Pennsylvania, United States

**Keywords:** neuroblastoma, histone acetylation, O-glycans, glycophenotype, MYCN

## Abstract

Neuroblastoma (NB) is the most common pediatric malignancy diagnosed before the first birthday in which MYCN oncogene amplification is associated with poor prognosis. Although aberrant glycosylation is an important actor in cell biology, little is known about its role in pediatric cancers such as NB. In this work we characterized the glycophenotype and the enzyme expression involved in glycans biosynthesis in five established human NB cell lines and in patient-derived primary tumors with different MYCN status. Our results show a high expression of Lewis glycan family both in MYCN-amplified cell lines and patient samples. Additionally, we report that MYCN-amplified cells overexpressed Core 2-initiating glycosyltransferase C2GNT1 in association with specific ST3Gals and FUTs, and showed increased binding to E- and P- selectins. Silencing of C2GNT1 expression in NB cells diminished expression of Lewis glycans, decreased the E- and P-selectin binding, and reduced cell adhesion, migration and proliferation *in vitro*. Treatment of MYCN-non-amplified cells with Trichostatin A (TSA), an histone deacetylase inhibitor, increased the expression of Lewis glycans and the enzymes involved in their biosynthesis. Our results demonstrate that MYCN-amplified NB cells overexpress Lewis family glycans, which belong to the Core 2 O-glycans group. Their expression plays a key role in the malignant behaviour of the NB cells and it is modulated by epigenetic mechanisms.

## INTRODUCTION

NB is the most common pediatric malignancy diagnosed before the first birthday and it is commonly localized in the abdomen [1]. It has a presumable cell origin in the sympathoadrenal lineage of the neural crest during development and represents about 6% of all cancers in children, with approximately 700 new cases appearing each year in the United States [2, 3].

The amplification of MYCN oncogene in sporadic NB is the most common focal genetic lesion. It occurs in approximately 22% of tumors and is associated with poor outcome [4, 5]. The International Neuroblastoma Risk Group Staging System (INRGSS) includes MYCN status together with age at diagnosis, histology and grade of tumor differentiation, presence or absence of 11q aberrations and tumor-cell ploidy to perform the actual risk-groups classification used in the clinic [6].

Despite the existence of intensive multimodal therapy and recent advances in target-directed therapies, which have improved NB patients´ responses, long-term survival of patients with high-risk NB remains poor. In this scenario, the glycolipid GD2 has demonstrated to be a good target for immunotherapy. In March 2015, the FDA approved a chimeric, human-murine, anti-GD2 monoclonal antibody known as Dinutuximab (Unituxin^©^, United Therapeutics Corporation, Silver Spring, MD) as an orphan drug designation for the maintenance treatment of pediatric patients with high risk NB. Dinutuximab achieved, as maintenance treatment in high risk NB, a partial response to first-line multiagent, multimodal therapy [7]. The clinical benefit of the anti-GD2 therapy for this indication stresses the important role that this glycolipid has in the biology of these tumor cells [8]. Nevertheless, many aspects of the participation of glycans in NB biology are still unknown.

In mammalian cells, glycans are made of at least 32 saccharides bound by different types of covalent bonds [9]. The participation of glycans in cell biology can be classified into four distinct categories that comprise 1) structural role, 2) inter-species recognition, 3) intra-species recognition and 4) molecular mimicry of host glycans [10]. In eukaryotic cells, glycans are attached to proteins defining O- and N-glycans branches. While O-glycans are mostly linked by an N-Acetylgalactosamine (GalNAc) to the hydroxyl group of the aminoacids threonine or serine, N-glycans are attached via the amide group of an asparagine residue [11, 12].

There are several reports describing the participation of glycans in cancer progression and dissemination [13, 14]. However, we are far from completely understanding the participation of glycans in cancer biology. Aberrant tumor glycosylation enables malignant cells to modulate the intra-species recognition, allowing them to escape from immune surveillance, promote inflammation, undergo epithelial-mesenchymal transition, increase vascular tumor cell-rolling and extravasation, and facilitate distant cell homing [15–17]. The altered glycophenotype in cancer involves different modifications in the cell surface, including falling or rising expression of certain glycans, accumulation of precursors and appearance of unfinished and novel structures [18]. The resulting glycophenotype depends on many factors, but mainly on the abnormal expression of the enzymes involved in the biosynthesis of glycan structures, also described as glycosyltransferases [19, 20].

Lewis blood group antigens are a related set of glycans with α1-3/α1-4 Fucose (Fuc) residues. It has been shown that Lewis glycans are overexpressed in different cancer indications as terminal glycan epitopes associated with bad prognosis and poor survival. In particular, Sialyl Lewis X (SLe^x^) and Sialyl Lewis A (SLe^a^) take part in the binding of E- and P- selectins to their ligands. Selectins mediate cellular extravasation and platelet aggregation that are highly associated with tumor cell circulation and dissemination. In certain tumors, mainly carcinomas, the overexpression of Lewis glycans on selectin ligands is reported to have an essential role in dissemination and metastasis [21, 22].

Even though our knowledge of glycan participation in tumor biology is growing faster, little is explored about the glycobiology of pediatric cancers and the role that glycans play in the malignant phenotype of tumors such as NB. Many reports are focused on the study of glycosyltransferases but very few describe glycan expression in this indication [23]. The first publication related to the expression of glycans in NB analyzed only 5 NB paraffin-fixed tumor samples, and described the loss of expression of the O-glycan blood-group-related antigens Thomsen–Friedenreich (T), Tn, Sialyl Tn (sTn), Le^a^, SLe^a^, Le^b^, Le^x^, SLe^x^, polyfucosyl Le^x^ and Le^y^ [24]. No other reports describing the general glycophenotype of NB can be found in the last years.

Regarding glycosyltransferases in NB, recent results published by Ho *et al.* associate favorable clinical prognosis with the expression of GALNT2, one of the enzymes that mediates the initial step of O-GalNAc glycosylation (GalNAcTs) [25]. The same group reported that overexpression of B3GNT3, which produces extended Core 1 O-glycans, down modulates the malignant phenotype in a NB cell line and predicts a favorable 5-year survival rate for NB patients [26]. In addition to GalNAcTs, Berois *et al.* proposed GALNT9 and GALNT13 as tumor prognostic markers in low and high risk tumors, respectively [27, 28]. Moreover, expression of GnT-V, an enzyme related to N-glycans biosynthesis, associates with a favorable prognosis and treatment outcome in NB patients. In the same report, it was demonstrated that GnT-V expression sensitizes NB cells to undergo apoptosis in response to retinoic acid [29].

Cancer glycobiology research has provided novel biomarkers and potential therapeutic targets in different cancer indications. In this work, we describe the glycan phenotype and the glycosyltransferases expression in a panel of NB cell lines and also in primary-tumor patient samples. Our results support the hypothesis that Lewis family glycans, as part of O-glycosylated proteins, have a role in the malignant phenotype of MYCN-amplified NB cells.

## RESULTS

The presence of Lewis glycan family (SLe^x^, Le^x^, SLe^a^, Le^a^, Le^y^ and Le^b^) and truncated O-glycans (Tn, STn and T) in human NB cell lines was evaluated by Flow Cytometry using specific mAb. Higher expression of Lewis glycans was observed in the MYCN-amplified cell lines (SK-N-BE (2), IMR-32 and CHP-212) in comparison with the non-amplified ones (SK-N-AS and SK-N-SH). Neither of the Lewis glycans showed high expression (greater than 1.5 rMFI) in MYCN-non-amplified cell lines. Conversely, we observed high or medium (between 1.25 and 1.5 rMFI) expression of SLe^x^, Le^x^, Le^y^ and Le^b^ in all the MYCN-amplified cell lines. Le^a^ and SLe^a^ expression was rated as medium and high in CHP-212 and SK-N-BE(2), respectively. Regarding truncated O-glycans, a low expression (lower than 1.25) was found in most of the cell lines evaluated. Only T antigen showed high and medium expression in SK-N-BE(2) and SK-N-SH, respectively (Table 1).

**Table 1:** Glycan expression evaluated by flow cytometry in the MYCN-amplified (SK-N-BE(2), IMR-32 and CHP-212) and MYCN-non-amplified (SK-N-AS and SK-N-SH) NB cell lines

	Neuroblastoma Cell Lines				
	MYCN-amplified			MYCN non-amplified	
**Lewis glycan family**	**SK-N-BE(2)**	**IMR-32**	**CHP-212**	**SK-N-AS**	**SK-N-SH**
**Slex**	1.87 + 0.14	1.32 + 0.09	5.33 + 1.29	1.23 + 0.13	1.15 + 0.09
**Lex**	2.19 + 0.63	1.51 + 0.09	1.66 + 0.14	1.16 + 0.16	1.05 + 0.10
**Slea**	1.10 + 0.18	1.03 + 0.01	1.32 + 0.31	1.09 + 0.08	1.11 + 0.05
**Lea**	1.53 + 0.25	1.08 + 0.05	1.01 + 0.08	1.17 + 0.05	1.03 + 0.12
**Ley**	1.75 + 0.45	1.72 + 0.55	2.70 + 0.30	1.04 + 0.11	1.15 + 0.13
**Leb**	2.29 + 0.30	2.27 + 0.19	1.32 + 0.20	1.37 + 0.06	1.05 + 0.02
**Truncated O-Glycans**					
**Tn**	1.06 + 0.02	1.07 + 0.02	1.07 + 0.05	1.06 + 0.04	1.15 + 0.06
**STn**	1.02 + 0.06	1.06 + 0.04	1.05 + 0.06	1.10 + 0.06	1.06 + 0.03
**T**	1.57 + 0.25	1.21 + 0.14	1.09 + 0.03	1.11 + 0.05	1.38 + 0.06

Data represent MFImeans ± S.D (rMFI) of triplicate determinants. High expression was considered greater than 1.5 rMFI, medium between 1.25 and 1.5 rMFI, and low as lower than 1.25 rMFI.

**Figure 1:**
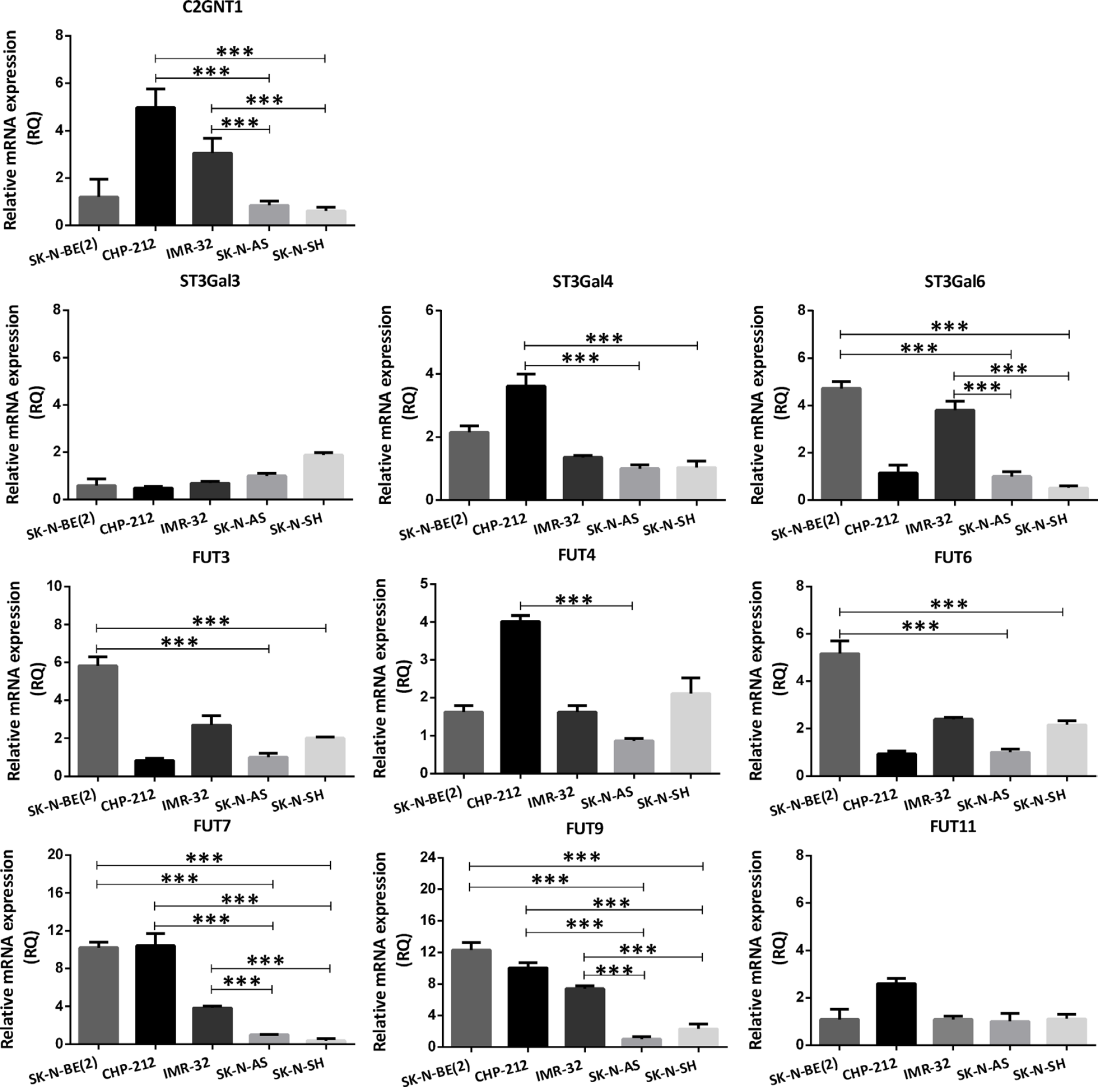
Comparison of glycosyltransferases transcripts expression involved in Core 2 O-glycan biosynthesis in NB cell lines The mRNA levels were analyzed using qRT-PCR. The relative amount of mRNA levels was normalized to the endogenous HPRT1 expression. A significant biological result was considered at threefold difference between samples values. Data represent means ± S.D. of three independent experiments (^***^p < 0.001, ANOVA followed by Tukey’s multiple comparisons test).

Transcription activity of the glycosyltransferases involved in the biosynthesis of Lewis antigens was carried out by qRT-PCR. Lewis glycans biosynthetic pathway is illustrated in Supplementary Figure 1. We evaluated the mRNA levels of the enzymes C2GNT1, which is responsible for the Core 2 structure in O-glycans, the sialyltransferases ST3GAL3/4/6 and the fucosyltransferases FUT3/4/6/7/9/11, which catalyze the addition of the sialic acid and the fucose residues on the Lewis family glycans, respectively. Regarding C2GNT1, two out of three MYCN-amplified cell lines showed more than threefold increase in mRNA expression compared with MYCN-non-amplified cells, in line with glycans expression. Although SK-N-BE(2) cell line did not overexpress C2GNT1, it shows overexpression of ST3Gal6 and FUT3/6/7/9. On the whole, all MYCN-amplified cell lines showed overexpression of at least one ST3Gal and more than one FUT (Figure 1). CHP-212 and SK-N-AS were chosen to conduct the rest of the experimental evaluations comparing MYCN-amplified and non-amplified cells, since they demonstrated the biggest difference in the glycophenotype profile.

**Figure 2:**
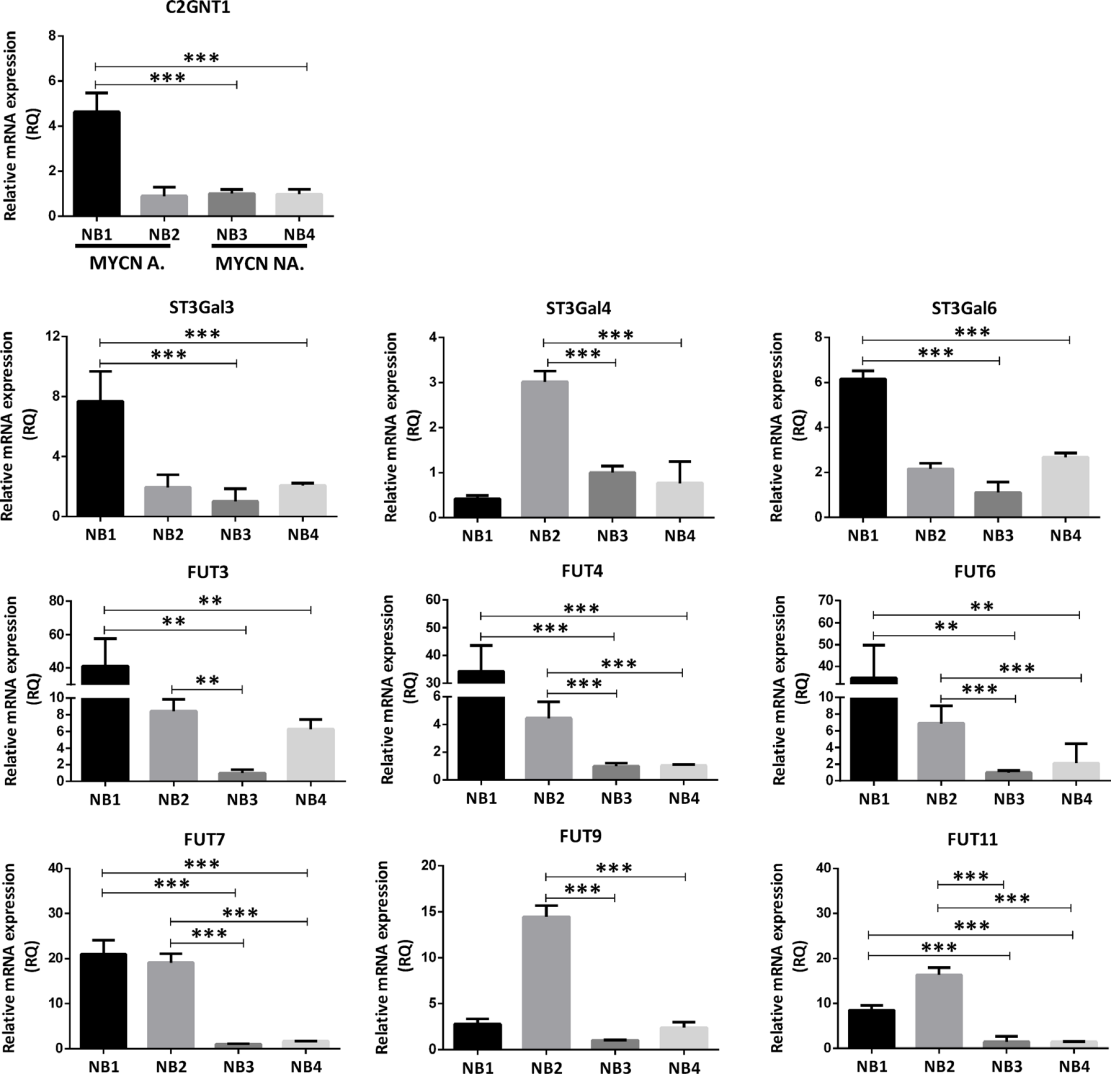
Comparison of glycosyltransferases transcripts expression involved in Core 2 O-glycan biosynthesis in patient-derived primary-tumor samples The mRNA levels were analyzed using qRT-PCR. The relative amount of mRNA levels was normalized to the endogenous HPRT1 expression. A significant biological result was considered at a threefold difference between samples values. Data represent means ± S.D. of three independent experiments (^**^p < 0.01, ^***^p < 0.001, ANOVA followed by Tukey’s multiple comparisons test).

Similar results were observed in the evaluation of the transcription level of these glycosyltransferases using primary-tumor samples from NB patients. Determination of MYCN status showed that two out of four NB were MYCN-amplified (NB1 and NB2) and the other two were non-amplified (NB3 and NB4). Transcription level of C2GNT1 was more than three times higher for NB1 in comparison with non-amplified tumor samples. Regarding downstream glycosyltransferases expression involved in the biosynthesis of Lewis glycans, NB1 showed higher levels of ST3GAL3/6 and FUT3/4/6/7/11. Even though C2GNT1 was not overexpressed in NB2, ST3GAL4 was overexpressed as well as all the FUTs evaluated (FUT3/4/6/7/9/11). MYCN-non-amplified samples NB3 and NB4 showed low glycosyltransferases transcription levels in comparison with the MCYN-amplified samples (Figure 2).

**Figure 3:**
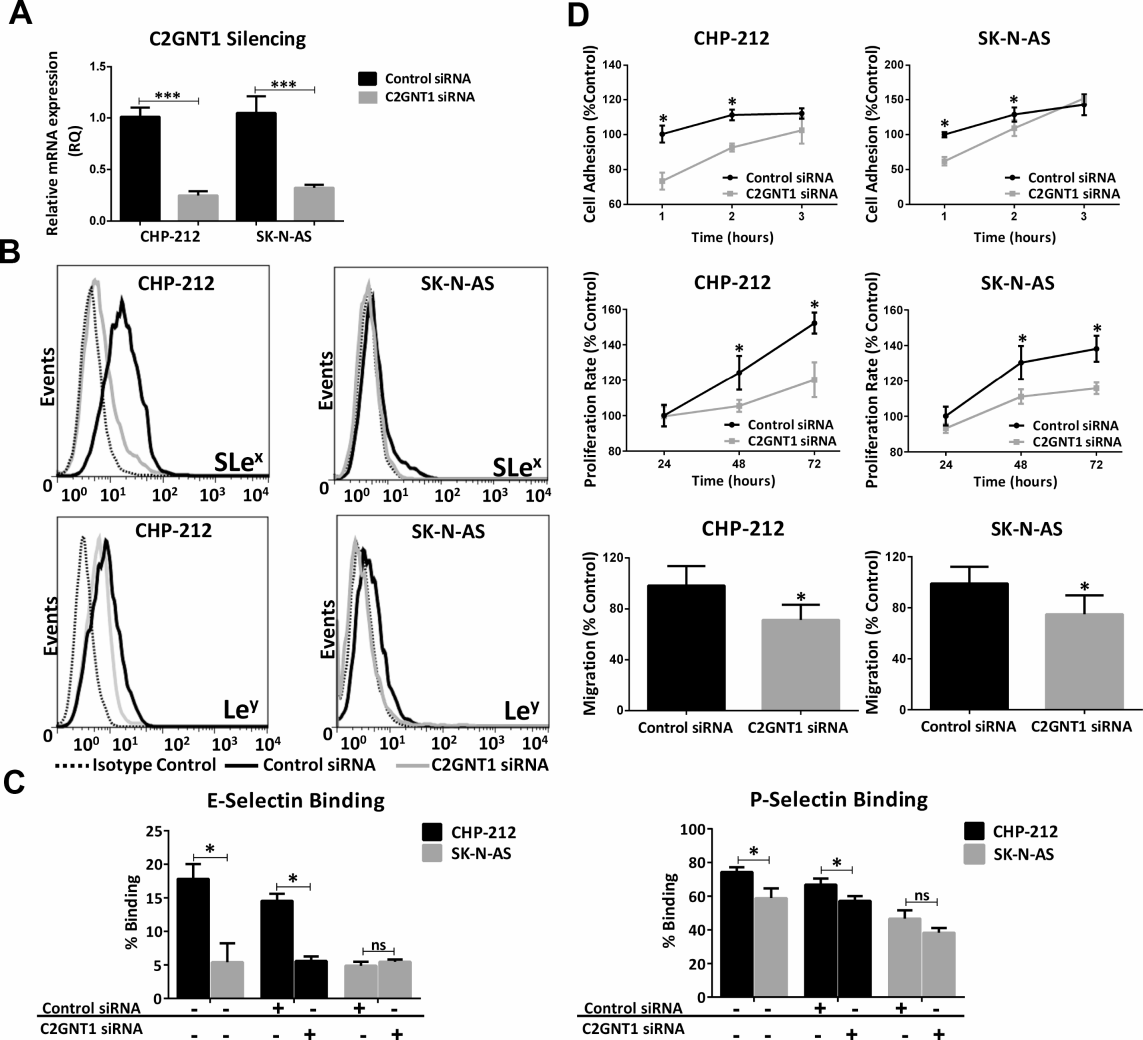
Silencing of C2GNT1 gene by siRNA technology in CHP-212 and SK-N-AS cell lines after 48 h of siRNA transfection (C2GNT1 and control) (**A**) Measurement of C2GNT1 transcript levels by qRT-PCR (^***^p < 0.001, *T*-test). (**B**) Analysis of SLe^x^ and Le^y^ expression. (**C**) Binding of selectins on control siRNA or C2GNT1 siRNA transfected cell lines and non-treated cells. (**D**) Cell adhesion measured at 1, 2 and 3 h; Cell proliferation measured at 24, 48 and 72 h (ANOVA followed by Tukey’s multiple comparisons post hoc test, ^*^p < 0.05); Cell migration capacity assessed by Transwell^®^ inserts (^*^p < 0.05, *T*-test). For all assays, data represent means ± S.D. of three independent assays.

In order to evaluate the participation of C2GNT1 expression in Lewis glycans biosynthesis, we treated CHP-212 and SK-N-AS cell lines with a specific small interfering RNA (siRNA). A significant decrease of C2GNT1 mRNA expression was obtained by qRT-PCR (Figure 3A). In addition, we observed a reduction in SLe^x^ and Le^y^ expression in these cells upon C2GNT1 silencing measured by Flow Cytometry (Figure 3B). To study if Lewis glycans were part of N-glycans we measured their expression after treatment with Tunicamycin (TNM). This treatment did not decrease expression of SLe^x^ and Le^y^ in any of the cell lines evaluated. As positive controls of TNM treatment, we measured Concanavalin A (ConA) or Phytohemagglutinin-L (L-PHA) binding, observing a decrease in the expression of N-glycans structures (Supplementary Figure 2).

Cell capacity to bind selectins or expression of selectin ligands was studied using Recombinant Human (E-, L- or P-) Selectin Fc chimeras. Our results showed higher binding to E- and P-selectin for CHP-212 in comparison with SK-N-AS. CHP-212 binding to E- and P- selectins decreased sharply due to C2GNT1 silencing (Figure 3C). No differences in L-selectin binding were found between both cell lines (data not shown). Additionally, higher expression of selectin ligand PSGL-1 was observed in CHP-212 compared to SK-N-AS (Supplementary Figure 3).

Furthermore, we observed a modulation in the *in vitro* cell behaviour when C2GNT1 was silenced. Our results show that cell adhesion, proliferation and also migration were significantly reduced by the decrease of C2GNT1 mRNA expression in CHP-212 and SK-N-AS cell lines. C2GNT1 knockdown provoked a significant reduction in adhesion, by almost 30% for CHP-212 and 35% for SK-N-AS cell line, compared with control siRNA transfected cells. Concerning cell proliferation, we observed a significant reduction when both cell lines were silenced at 24 h. Moreover, cell migration significantly decreased after C2GNT1 knockdown, in CHP-212 as well as in SK-N-AS (Figure 3D).

**Figure 4:**
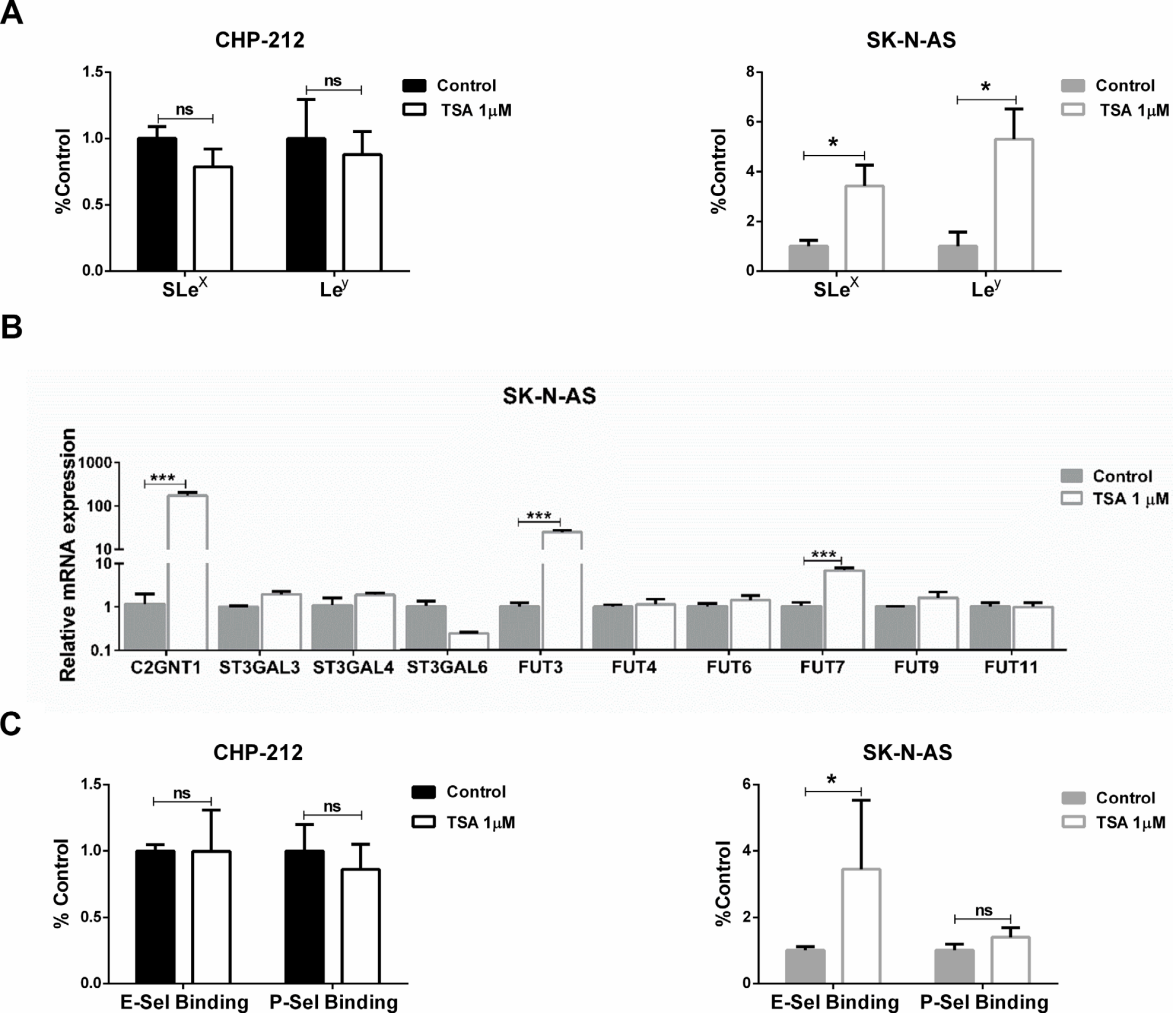
Evaluation of epigenetic regulation CHP-212 and SK-N-AS cell lines were treated with TSA 1 mM for 24 h and controls in DMSO. (**A**) SLe^x^ and Le^y^ expression (^*^p < 0.05, *T*-test). (**B**) Glycosyltransferases mRNA expression (^***^p < 0.001, *T*-test). (**C**) Binding of E-, P-selectin (^*^p < 0.05, Mann–Whitney for E-selectin binding and *T*-test for P-selectin binding). Data represent means ± S.D. of three independent assays.

**Figure 5:**
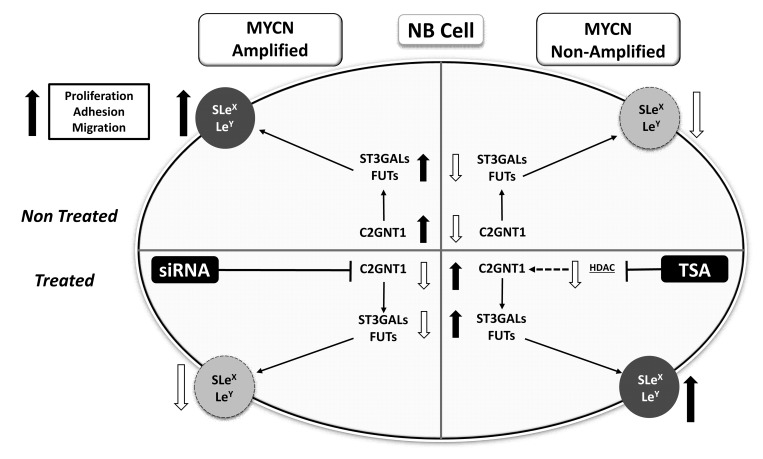
Schematic representation of results obtained for Lewis glycans and glycosyltransferases expression in treated and untreated MYCN-amplified and non-amplified NB cells

Finally, we wondered if the observed glycophenotype was regulated by epigenetic mechanisms. To answer this, we treated the cells with Trichostatin A (TSA) and as a consequence we found a significant increase in the expression of SLe^x^ and Le^y^ in treated SK-N-AS cells. No expression changes were recorded in TSA-treated CHP-212 cells (Figure 4A). The presence of Lewis glycans in MYCN-non-amplified cells after TSA treatment accounted for a significant increase in the transcription rate of C2GNT1 and FUT3/7 (Figure 4B). Moreover, our results also show a significant increase in E-selectin ligands expression in MYCN-non-amplified cells treated with TSA (Figure 4C). Considering all the results obtained in this work, we proposed a hypothetical functional model described in Figure 5.

## DISCUSSION

Proteins and lipids-associated carbohydrates have demonstrated to play a crucial role in the interaction between cancer cells and tumor microenvironment. Most of our prevailing knowledge about cancer glycosylation has been obtained working on adult indications. Conversely, much less is known regarding pediatric cancers such as NB. Our work describes the expression of Lewis antigens in the context of O-glycans in NB cells and the consequences of their downregulation in cell behaviour. These results support the hypothesis that this group of glycans plays an important role in NB cell malignancy.

Abnormal synthesis of cancer-associated glycans occurs as a consequence of the deregulated expression of glycosyltransferases genes. While sialyltransferases catalyze the transference of a sialic acid (Sia) from CMP-Sia to the hydroxyl group at the terminal non-reducing position of Galactose, N-Acetylglucosamine (GlcNAc), GalNAc or Sia monosaccharide, fucosyltransferases are responsible for the transference of an L-fucose sugar from GDP-fucose to an acceptor substrate [30]. The biosynthesis of Lewis glycans depends on the expression of enzyme members of one or both of these gene families. In adult cancers, such as breast and colorectal cancer, it has been demonstrated that overexpression of ST3Gal3 and FUT4 is associated with poor prognosis [31–33].

The glycophenotype characterization of five established and validated NB cell lines revealed high expression of the Lewis family glycans (Le^x^, SLe^x^, Le^y^ and Le^b^) in those with amplification of the oncogene MYCN. Our results show that high expression of the Lewis antigens in MYCN-amplified cell lines could be a consequence of the overexpression of ST3Gal4/6 and more than one FUT. Interestingly, ST3Gal3 and FUT11 were not overexpressed in any of the cell lines evaluated. No glycosyltransferases overexpression was detected in MYCN-non-amplified cell lines in accordance with the low expression of glycans. Similar results were obtained with patient-derived primary-tumor samples; while overexpression of ST3Gal3/6 was detected in one MYCN-amplified patient sample (NB1), overexpression of ST3Gal4 was found in the other (NB2). In MYCN-amplified patient samples, we also found raised transcript levels in most of the FUTs. In relation to this, it is reported that several FUTs have overlapping roles in this metabolic route [34–36]. Our findings suggest that, in the biosynthesis of Lewis glycans, redundant enzymatic activities seem to be equally chosen by malignant cells.

The enzyme C2GNT1 synthesizes the Core 2 O-glycans by catalyzing the bond of GlcNAc to the Core 1, being the only enzyme capable of this activity. The higher gene transcription of C2GNT1 in MYCN-amplified IMR-32 and CHP-212 in contrast to the MYCN-non-amplified SK-N-SH and SK-N-AS cell lines, suggests that Lewis glycans are expressed as part of Core 2 O-glycans in MYCN-amplified cells. Overexpression of C2GNT1 mRNA levels was also observed in NB1 MYCN-amplified patient tumor sample. Although the other MYCN-amplified tumor sample NB2 showed low transcription levels of C2GNT1, we observed remarkable high levels of most of the downstream glycosyltransferases evaluated, suggesting a presumed high expression of Lewis glycans. Evaluation of Lewis glycans presence should be conducted on patient samples to analyze their association with MYCN status. Subsequent experiments in cell lines showed a significant reduction in the expression of SLe^x^ and Le^y^ when C2GNT1 expression was silenced. In this line, no changes in the expression of SLe^x^ and Le^y^ were observed as a result of TNM treatment, confirming the low presence of these antigens in N-glycans. Moreover, silencing of C2GNT1 diminished *in vitro* cell adhesion, proliferation and migration indicating the participation of O-glycans in these cell behaviors.

Ho *et al.* recently published that overexpression of GALNT2, one of the GalNAcTs involved in O-glycans biosynthesis, suppresses malignant phenotype by increasing the amount of T and Tn on the IGF-1 receptor [25]. It is described in another publication that overexpression of B3GNT3, the enzyme that is responsible for adding GlcNAc to Core 1 to produce Extended Core 1 oligosaccharides, down modulates the malignant phenotype including migration and invasion [37]. Additionally, B3GNT3 has been proposed as an independent prognostic factor of better survival outcome after the evaluation of its expression in NB tumor tissues [26]. Considering that B3GNT3 and C2GNT1 use the same substrate but lead to a different glycan branching with a different role in malignant cell behavior, it can be proposed that Ho *et al.* results are complementary to this work supporting the hypothesis that extended Core 1 formation down modulates the malignant phenotype while Core 2 formation promotes it.

Hill and colleagues reported that the expression of SLe^x^ in the context of Core 2 O-glycan regulates invasion by E-selectin ligands, using Colon and Hepatic Carcinoma cell lines [38]. In our work, we showed that the MYCN-amplified cell line CHP-212 presents high levels of SLe^x^, catalyzed by ST3Gal4 and FUT4/7/9 in the context of Core 2 O-glycans. Furthermore, in the evaluation of total selectin ligands by selectin Fc quimera in both cell lines, we also observed higher binding of E- and P- selectins to the MYCN-amplified cell line. The silencing of C2GNT1 significantly reduced E- and P-selecting binding. Selectins have a wide panel of ligands in which Lewis glycans have a key role. Post-translational modification of P-selectin ligands with SLe^x^ was reported in NB cell lines among other indications, but their association with C2GNT1 expression was not reported [39]. Additionally, CHP-212 shows higher expression of the natural ligand PSGL-1.

It is known that MYCN cannot initiate *de novo* gene transcription but is a key element in the recruitment of nucleosome-modifiers, mainly histone acetyltransferases (HAC), diminishing histone deacetyltransferases (HDAC) to promote a positive transcription landscape [40]. Abnormal epigenetic regulation involving HDACs has been largely associated with cancer development. HDACs are a family of 18 enzymes that maintain histones in a positively charged state which in turn promotes a condensed chromatin structure that prevents gene transcription. A large body of evidences indicates that HDACs are involved in cancer progression at cell cycle regulation, apoptosis, DNA damage response, metastasis and angiogenesis [41]. HDACs participation on NB has been previously reported. A gene expression analysis published by Keshelava *et al.* on 17 cell lines demonstrated a strong association of HDAC1 gene expression with multidrug resistance [42]. In addition, a correlation between the HDAC8 expression, disease stage and poor outcome was published by Oehme *et al.* analyzing 118 NB patient samples by q-RT-PCR. In this work, the authors showed a correlation between HDAC8 expression and the International Neuroblastoma Staging System (INSS) stages, as well as with the Shimada pathology classification among others, but they were unable to report an association with MYCN amplification status [43]. Very few reports stand on the relation between HDACs, glycophenotype and glycosyltransferases regulation as it is reported in this work. The incubation of SK-N-AS cells with the broad spectrum HDACs inhibitor TSA promotes a remarkable overexpression of C2GNT1 and FUT3/7 which results in a high expression of Le^y^ and SLe^x^. Consequently, the modification of the glycophenotype on these MYCN-non-amplified cells increases the presence of selectin ligands.

The results presented in this work described the biosynthetic route and the epigenetic regulation involved in the expression of Lewis family antigens as part of Core 2 O-glycans. In summary, our results and the evidence provided by other groups strongly support the hypothesis that Lewis family glycans as part of Core 2 O-glycans play a relevant role in NB malignant cell behavior in MYCN-amplified cells.

## MATERIALS AND METHODS

### Cell lines

Human MYCN-amplified NB cell lines SK-N-BE(2) (CRL-2271), IMR-32 (CCL-127) and CHP-212 (CRL-2273), and human MYCN-non-amplified NB cell lines SK-N-AS (CRL-2137) and SK-N-SH (HTB-11) were obtained from the American Type Culture Collection (ATCC, Virginia, United States) [44, 45]. SK-N-BE(2) and CHP-212 cells were grown in a mixture (1:1) of Eagle’s Minimum Essential Medium (Sigma Aldrich by Merck, Darmstadt, Germany) and Ham´s F-12 Nutrient Mix (Gibco, California, United States). SK-N-AS cells were grown in Dulbecco’s Modified Eagle’s Medium (Gibco, California, United States) and 0.1 mM of Non-Essential Amino Acids (Gibco, California, United States). IMR-32 and SK-N-SH cells were grown in Eagle’s Minimum Essential Medium. 10% fetal bovine serum (FBS) (Gibco, California, United States) was added and the cell cultures were maintained at 37° C in a humidified atmosphere of 5% CO_2_. Cell lines were tested for *Mycoplasma* every three months by DAPI staining (Vector, California, United States). Cells passages lower than 20 were used in the described experiments.

### Patient-derived primary-tumor samples

Different amounts (1 to 2 mg) of four samples of human pediatric NB tumors were chosen for RNA extraction. Two samples presented MYCN-amplified (NB1 and NB2) and two MYCN non-amplified status (NB3 and NB4) evaluated by IHC (data not shown). Tumor samples were obtained from the Pediatric Hospital “Prof. Dr. Juan P. Garrahan”. MYCN status was determined by interfering FISH technique with Vysis Paraffin II Pretreatment Reagent Kit (Abbott Laboratories, Illinois, United States) on histological FFPE sections of 3 μm thickness according to the manufacturer›s instructions. Briefly, the deparaffinisation of the histological section was performed with xylene, then a pretreatment with a guanidinium thiocyanate solution for 10 min at 80° C was performed followed by enzymatic digestion at 37° C for 10 min. After that, tissue sections were incubated with LSI MYCN probe (2p24.1) in the hybridizer HYBrite (Abbott Laboratories, Illinois, United States) for co-denaturation (73° C for 5 min) and hybridization (37° C for 20 h). Post-hybridation washes were performed using 2× SSC buffer with NP40. Tissue sections were stained with DAPI and observed with a fluorescence microscope (Eclipse 80i, Nikon, Tokyo, Japan). Patients gave their consent to perform the proposed diagnostic and therapeutic procedures, and everything necessary to achieve them. The project was approved by the Research Ethics Committee and the Research Review Committee of the Argentinean Pediatric Hospital “Prof. Dr. Juan P. Garrahan”.

### Flow cytometry assay

After 24 h of serum starvation, cell lines were collected by enzyme-free cell dissociation buffer (Gibco by Thermo Fisher Scientific, California, United States) and incubated with antigens specific mAbs or isotype control for 30 min on ice. The mAbs used were Le^x^ (4E10, Novus Biologicals, Colorado, United States), SLe^x^ (CSLEX1, BD Pharmingen, California, United States), Le^a^ (Abcam, Cambridge, United Kingdom), SLe^a^ (KM231, Millipore, Massachusetts, United States), Le^b^ (2-25LE, Abcam, Cambridge, United Kingdom), Le^y^ (F3, Abcam, Cambridge, United Kingdom), PSGL-1 (PL-1, eBioscience by Thermo Fisher Scientific, California, United States), T (Thomsen–Friedenreich Antigen, SPM320, Novus Biologicals, Colorado, United States), Tn (Tn 218, Abcam, Cambridge, United Kingdom), STn (B72.3, Santa Cruz, Texas, United States), Mouse IgG1 Isotype Control (Thermo Fisher Scientific, California, United States), Mouse IgM Isotype Control (Dako, Santa Clara, United States). Cells were washed with phosphate buffered saline (PBS) and incubated with Polyclonal Goat Anti-Mouse Phycoerythrin (PE) labeled anti-mouse immunoglobulins (Dako, Santa Clara, United States) for 30 min on ice. For lectins binding, biotinylated ConA or L-PHA (Vector Laboratories, United States) were incubated for 30 min on ice with fluorescein streptavidin to form a complex. Then, cells were incubated 30 min on ice with the lectin-streptavidin complex. For mAbs or lectins, acquisition was achieved by a FACSCalibur flow cytometer (Becton Dickinson, New Jersey, United States) and analyzed by the FlowJo^®^ software. Data was analyzed as median florescence intensity relativized to isotype control (rMFI). High expression of terminal glycan structures (Lewis and truncated) was defined as greater than 1.5 rMFI, medium: between 1.25 and 1.5 rMFI, and low as lower than 1.25 rMFI.

### Selectin binding assay

CHP-212 and SK-N-AS cell lines were collected by enzyme-free cell dissociation buffer and incubated with selectin complexes for 30 min on ice, washed and analyzed using a FACSCalibur (Becton Dickinson, New Jersey, United States) and analyzed by the FlowJo^®^ software. Production of complexes was carried out in PBS buffer + 1 mM Ca^2+^ + 1 mM Mg^2+^ with 1 mg/ml of Recombinant Human (E-, L- or P-) Selectin Fc chimera (R&D systems, Minnesota, United States) or Fc control (R&D systems, Minnesota, United States) and 0.5 mg/ml of goat anti-human-IgG-FITC (Abcam, Cambridge, United Kingdom).

### Quantitative RT-PCR (qRT-PCR)

Total RNA from cell lines after 24 h of serum starvation (1 × 10^6^ cells) or frozen samples of NB patients, was purified with the PureLink^®^ RNA Mini Kit (Invitrogen, by Thermo Fisher Scientific, California, United States) according to the manufacturer’s protocol. RNA was reverse transcribed with Super Script™ III (Thermo Fisher Scientific, California, United States) according to the manufacturer’s protocol. The concentration and purity of RNA was assessed using a Nanodrop spectrometer (Thermo Fisher Scientific, California, United States). Primers were designed using the Primer3 plus software (http://www.bioinformatics.nl/cgi-bin/primer3plus/primer3plus.cgi) and checked for their specificity by The Basic Local Alignment Search Tool (BLAST). Primers were manufactured by Invitrogen (Invitrogen, by Thermo Fisher Scientific, California, United States) and their sequences are provided in Supplementary Table 1. qRT-PCR was performed using Power SYBR™ Green PCR Master Mix (Thermo Fisher Scientific, California, United States) and StepOne Real-Time PCR System (Applied Biosystems, California, United States). The following thermal cycling conditions were used: 48° C for 30 min, 95° C for 10 min, 40 cycles of 95° C for 15 seconds followed by 60° C for 60 seconds. Each sample was analyzed in triplicate and mean cycle threshold values (Ct) were used for further analysis. Ct values were normalized for hypoxanthine phosphoribosyltransferase 1 (HPRT1) expression levels. Relative quantification (RQ) values were calculated as 2^−ΔΔCt^. A significant biological result was assumed when a threefold difference between samples values was obtained.

### siRNA mediated knockdown

C2GNT1 expression was downmodulated by using siRNA. C2GNT1 and a scramble siRNAs (control) were obtained from Origene (Maryland, United States). C2GNT1 construct is composed by 3 unique 27mer siRNA duplexes (Locus ID 2650), and control siRNA is a Trilencer-27 Universal Scrambled Negative Control. Cells were plated on 60 mm dishes, grown until approximately 80% of confluency and then transfected with C2GNT1 siRNA or control siRNA using Lipofectamine 2000 (Thermo Fisher Scientific, California, United States) following manufacturer’s instructions. 24 h after transfection, cells were collected and plated for adhesion and proliferation assays or 48 h after transfection cells were processed for qRT-PCR.

### Tunicamycin (TNM) treatment

Cells were cultured in the absence or in the presence of 150 nM TNM from *Streptomyces sp.*(Merck KGaA, Darmstadt, Germany) for 24 h, controls were cultured in the presence of an equivalent volume of DMSO [46]. After treatment, presence of SLe^x^, Le^y^ and N-glycan branching was measured by flow cytometry.

### Trichostatine A (TSA) treatment

TSA was kindly provided by Dr. Norberto W. Zwirner, Laboratory of Pathophysiology of Innate Immunity, Institute of Experimental Biology and Medicine (IBYME), Argentina. Cells were cultured in the presence of 1 mM TSA for 24 h, controls were cultured in the presence of an equivalent volume of DMSO [47]. After treatment, SLe^x^/ Le^y^ expression and E-, P-selectin binding were measured by flow cytometry.

### Cell proliferation assay

Proliferative effect of C2GNT1 silencing was measured on rapidly growing tumor cells using MTS assay (3-(4, 5-dimethylthiazol-2-yl)-5-(3-carboxymethoxyphenyl)-2-(4-sulfophenyl)-2H-tetrazolium; Promega, Madison, United States). Cells were plated in 96-well flat bottom plates at a density of 5 × 10^3^ cells/well in 200 µl of corresponding medium supplemented with 10% FBS. MTS reagent (20 µl) was added to each well and the plate was incubated at 37° C for 2–4 hours. The absorbance of each well was measured in a microplate reader at 490 nm. The proliferative effect was measured at 24 h, 48 h and 72 h.

### Cell adhesion assay

Cell adhesion was measured by a colorimetric method, as previously reported [48]. CHP-212 and SK-N-AS cells, after 48 h of siRNA transfection (C2GNT1 or control) were harvested with an enzyme-free cell dissociation buffer and seeded at a concentration of 4 × 10^4^ cells/well in complete medium in a 96-well plate. After 1–3 h of incubation at 37° C, cells were washed with PBS, and non-adherent cells were removed by aspiration. Adherent cells were stained with 0.5% violet crystal diluted in 20% methanol and rinsed with distilled water. The dye in the stained cells was solubilized by adding 10% methanol 5% acetic acid solution, and the absorbance was measured at 595 nm using a microplate reader. Data was expressed as percentage of the control.

### Cell migration assay

After overnight starvation, 3 × 10^5^ CHP-212 cells or 3 × 10^3^ SK-N-AS cells previously transfected for 48h with siRNA were seeded into the Transwell^®^ inserts with 8 µm pore (Corning, New York, United States) in serum-free medium. The lower chamber was filled with medium containing 10% FBS as chemoattractant. Stationary cells were removed from the upper surface of the membranes with a cotton swab. Cells that migrated to the lower surface were fixed and stained with violet crystal. Migrating cells in five randomly selected fields were counted and normalized to control.

### Statistical analysis

Statistical significance was evaluated using Prism 6 statistical software (GraphPad, Inc. California, United States). Results presented in this study are expressed as mean values ± SD. For comparisons between two independent samples, *T*-test or Mann–Whitney was used. For multiple comparisons between experimental groups ANOVA (followed by Tukey’s multiple comparisons test) was performed. Data correspond of at least three independent experiments. Significant levels were defined as *p* < 0.05.

## SUPPLEMENTARY MATERIALS FIGURES AND TABLE



## Abbreviations

C2GNT1: Core 2 Beta1,6 N-Acetylglucosaminyltransferase
FDA: Food and Drug Administration
FUT: fucosyltransferase
GalNAc: N-Acetylgalactosamine
GalNAcTs: N-Acetylgalactosaminetransferases
GlcNAc: N-Acetylglucosamine
HDACs: histone deacetylases
HPRT1: hypoxanthine phosphoribosyltransferase 1
INRGSS: International Neuroblastoma Risk Group Staging System
Le^a^: Lewis A
Le^b^: Lewis B
Le^x^: Lewis X
Le^y^: Lewis Y
MYCN: Neuroblastoma MYC Oncogene
NB: Neuroblastoma
PBS: phosphate buffered saline
PE: Phycoerythrin
qRT-PCR: Quantitative RT-PCR
rMFI: relative Median Florescence Intensity
RQ: Relative quantification
Sia: sialic acid
siRNA: Small interfering RNA
SLe^a^: Sialyl Lewis A
SLe^x^: Sialyl Lewis X
ST: sialyltransferase
STn: SialylTn
TNM: Tunicamycin
TSA: Trichostatin A
T: Thomsen–Friedenreich Antigen
