# Supplementary material for: Aberrant O-glycosylation modulates aggressiveness in neuroblastoma
Source: Oncotarget. 2018 Sep 25;9(75):34176–88. doi: 10.18632/oncotarget.26169 (PMC6183345; doi:10.18632/oncotarget.26169)
Supplement: Supplementary file 1 [file oncotarget-09-34176-s001.pdf]

Aberrant O-glycosylation modulates aggressiveness in neuroblastoma

SUPPLEMENTARY MATERIALS

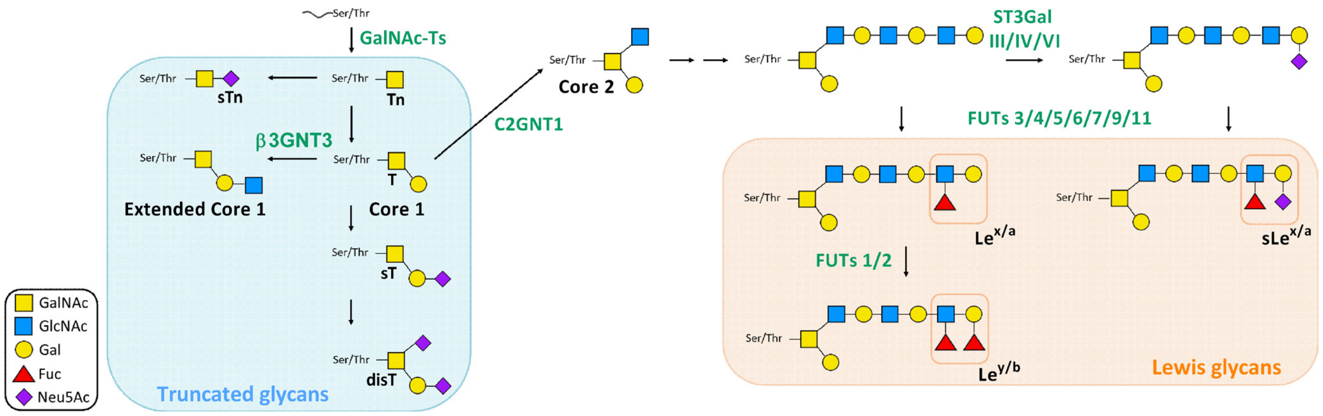

Supplementary Figure 1: Biosynthetic pathway of Lewis family in O-glycans branching.

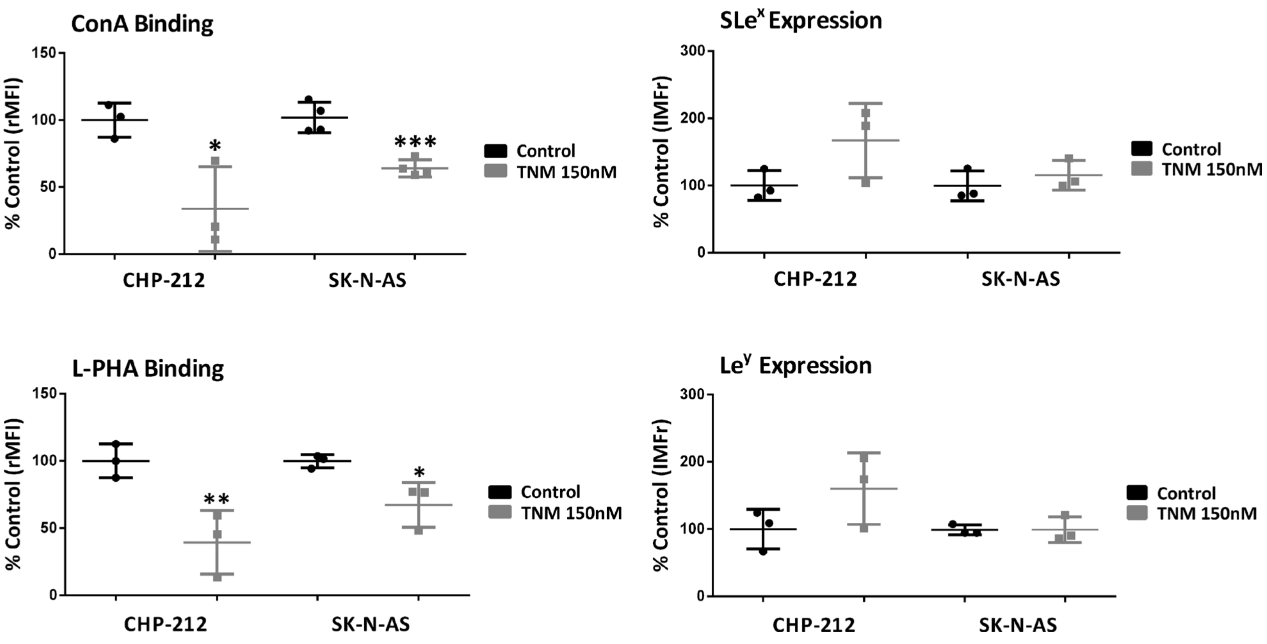

Supplementary Figure 2: Evaluation of ConA and L-PHA binding of CHP-212 and SK-N-AS as positive control of TNM treatment (150 nM). Evaluation of Sle^x and Le^y expression after TNM treatment. Data represent rMFImeans ± S.D. of triplicate determinants (ns $p > 0.05$, $^{*}p < 0.05$, $^{**}p < 0.001$, T-test.).

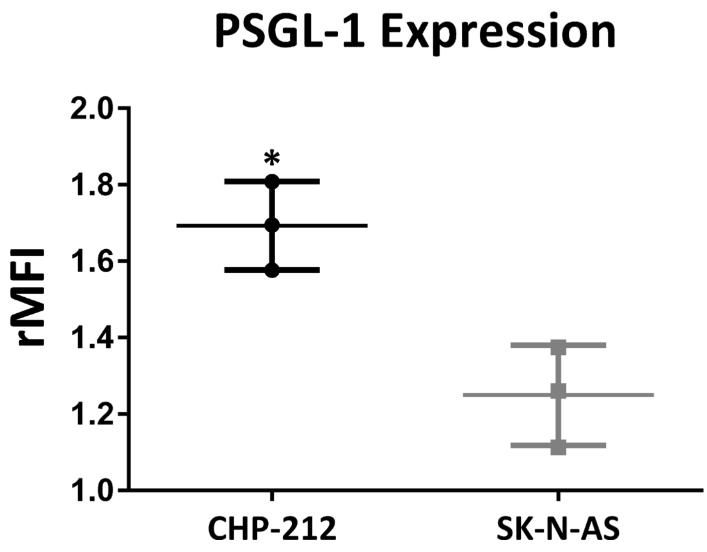

Supplementary Figure 3: PSGL1 expression by FACS. Data represent rMFI means ± S.D. of triplicate determinants ($^{*}p < 0.05$, T-test).

Supplementary Table 1: Sequence of the primers used for the analysis of enzymes by quantitative RT-PCR

Gene	Forward	Reverse
**HPRT1**	AACGTCTTGCTCGAGATGTG	GCTTTGATGTAATCCAGCAGG
**C2GNT1**	AAGCAGTTGCCAGGTTTG	ACACTGAGCGCACATGGAC
**ST3Gal3**	TCTCCGCTGTGGTCATTTAGG	AGTACCAGAAAGAGGCAGAGG
**ST3Gal4**	AGTAGAAAACAACCCAGACAC	AGAGGTTGAGAATCCGAA
**ST3Gal6**	AGAGTCCTTTGCACTACTATGG	CACTGTTAGCATCATCTTCTGAG
**FUT3**	GGGTTAAGAGCTCAGAGTTCAGAC	AGCAGCAATTTCCCTCAACCC
**FUT4**	TTGCACAGCTAGCAATTGGG	ATTCAGGAAACCGCCTCAAC
**FUT6**	AAGCCACATCGCATTGAAGC	TGGAGCCCGGACATCCTTTG
**FUT7**	CACCTCCGAGGCATCTTCAACTG	CGTTGGTATCGGCTCTCATTCATG
**FUT9**	CTTACCGCCGTGATTCAGAT	AATGCTTGCCCGTAGGTATG
**FUT11**	CTCTTGGCTTTCTTGTCC	ATGACGGAGTGATTGTTC

HPRT1 was used as endogenous control.
